# Expansion of human midbrain floor plate progenitors from induced pluripotent stem cells increases dopaminergic neuron differentiation potential

**DOI:** 10.1038/s41598-017-05633-1

**Published:** 2017-07-20

**Authors:** Stefania Fedele, Ginetta Collo, Katharina Behr, Josef Bischofberger, Stephan Müller, Tilo Kunath, Klaus Christensen, Anna Lisa Gündner, Martin Graf, Ravi Jagasia, Verdon Taylor

**Affiliations:** 1 Department of Biomedicine, University of Basel, Mattenstrasse 28, CH-4058 Basel, Switzerland; 2 Department of Molecular and Translational Medicine, Viale Europa 11, 25123 Brescia, Italy; 3 Department of Biomedicine, University of Basel, Pestalozzistrasse 20, CH-4056 Basel, Switzerland; 4 Pharma Research & Early Development, Roche Innovation Center Basel, F. Hoffmann-La Roche, Grenzacherstrasse 124, CH-4070 Basel, Switzerland; 5 MRC Centre for Regenerative Medicine, University of Edinburgh, 5 Little France Drive, EH93JQ Edinburgh, United Kingdom

## Abstract

Human induced pluripotent stem cells (hiPSCs) are invaluable to study developmental processes and disease mechanisms particularly in the brain. hiPSCs can be differentiated into mature and functional dopaminergic (DA) neurons. Having robust protocols for the generation of differentiated DA neurons from pluripotent cells is a prerequisite for the use of hiPSCs to study disease mechanisms, for drug discovery, and eventually for cell replacement therapy. Here, we describe a protocol for generating and expanding large numbers of homogeneous midbrain floor plate progenitors (mFPPs) that retain efficient DA neurogenic potential over multiple passages and can be cryobanked. We demonstrate that expanded mFPPs have increased DA neuron potential and differentiate more efficiently and rapidly than progenitors generated by standard protocols. In addition, this novel method results in increased numbers of DA neurons that *in vitro* show characteristic electrophysiological properties of nigrostriatal DA neurons, produce high levels of dopamine, and integrate into host mice when grafted *in vivo*. Thus, we describe a robust method for producing human mesencephalic DA neurons from hiPSCs.

## Introduction

Human pluripotent stem cells, including embryonic stem cells and hiPSCs, can be expanded *in vitro* and retain their capacity to differentiate into any cell type of the three germ layers^1, 2^. Hypothetically, they represent an unlimited source of cells for several applications including drug screening and cell replacement therapy for treatment of neurological disorders. Parkinson´s disease (PD), the second most common neurodegenerative disorder, is characterized by the selective loss of DA neurons of the substantia nigra of the midbrain^3, 4^. Although recent advances have been made in our understanding of the pathogenesis of PD, at present there are no cures and the main treatment for patients are DA analogues and receptor agonists to counteract the reductions in DA^5–7^. Numerous protocols have been developed to generate human DA neurons *in vitro* from pluripotent cells^8–14^. These methods rely on the directed differentiation of pluripotent cells using small molecules and growth factors either through an embryoid body or neurosphere step or in adherent culture^8–18^. These procedures are often laborious, long and highly variable resulting in heterogeneous differentiation with relatively low numbers of midbrain DA neurons. In order to use hiPSC-derived DA neurons for addressing gene function, for drug screening or eventual cell replacement therapy, a homogeneous, robust and rapid method would be a significant advantage^19^. Here we describe a robust and reproducible method that takes advantage of the mFPP-based differentiation strategy to generate cultures of ~100% LMX1A^+^FOXA2^+^ mFPP cells that can be expanded and maintained as a pure population^11^. This method takes advantage of the molecular pathways that guide DA neuron formation *in vivo* and can be used to generate a large number of mFPPs that can be passaged more than 6 times *in vitro* while retaining DA neuron differentiation potential. We also show that expanded mFPPs can be frozen and thawed and that they generate mature DA neurons *in vitro* with higher efficiency than those generated by standard protocols. Therefore, this protocol allows for expansion and banking of expanded mFPP for large-scale generation of mature and functional DA neurons *in vitro* and *in vivo* and circumvents some of the variability often seen with protocols that require differentiation from pluripotent cells without the possibility of progenitor expansion. The generation of expandable mFPPs on a large scale also makes this protocol advantageous for understanding cellular and molecular mechanisms of early human DA neuron development, generating large numbers of mFPPs or DA neurons for drug screening, and transplantation.

## Results

### Human iPSC-derived mFPPs can be expanded *in vitro*

Many DA neuron differentiation methods have been developed using human pluripotent stem cells as a cell of origin. Some protocols require the differentiation of cells via a embryoid body, 3D culture or neurosphere step^14, 20^ or induction of a pan-neural progenitor cell state^21–23^ for the formation of primitive neuroepithelia and neuronal rosette-like structures followed by ventralization to a mesencephalic fate. However, a mixture of dorsal and ventral neural subtypes are usually generated in these cultures including DA neurons, motor neurons, astrocytes and oligodendrocytes^21, 24, 25^. mFPP-based differentiation strategies were described in which sequentially factor application to the iPSCs results in directed ventralization and the generation of mFPPs^10, 11, 26–28^. The mFPPs are committed precursors which generate mesencephalic DA neurons *in vivo*. None of these methods take advantage of a DA neuron-specified progenitor cell population.

**Figure 1:**
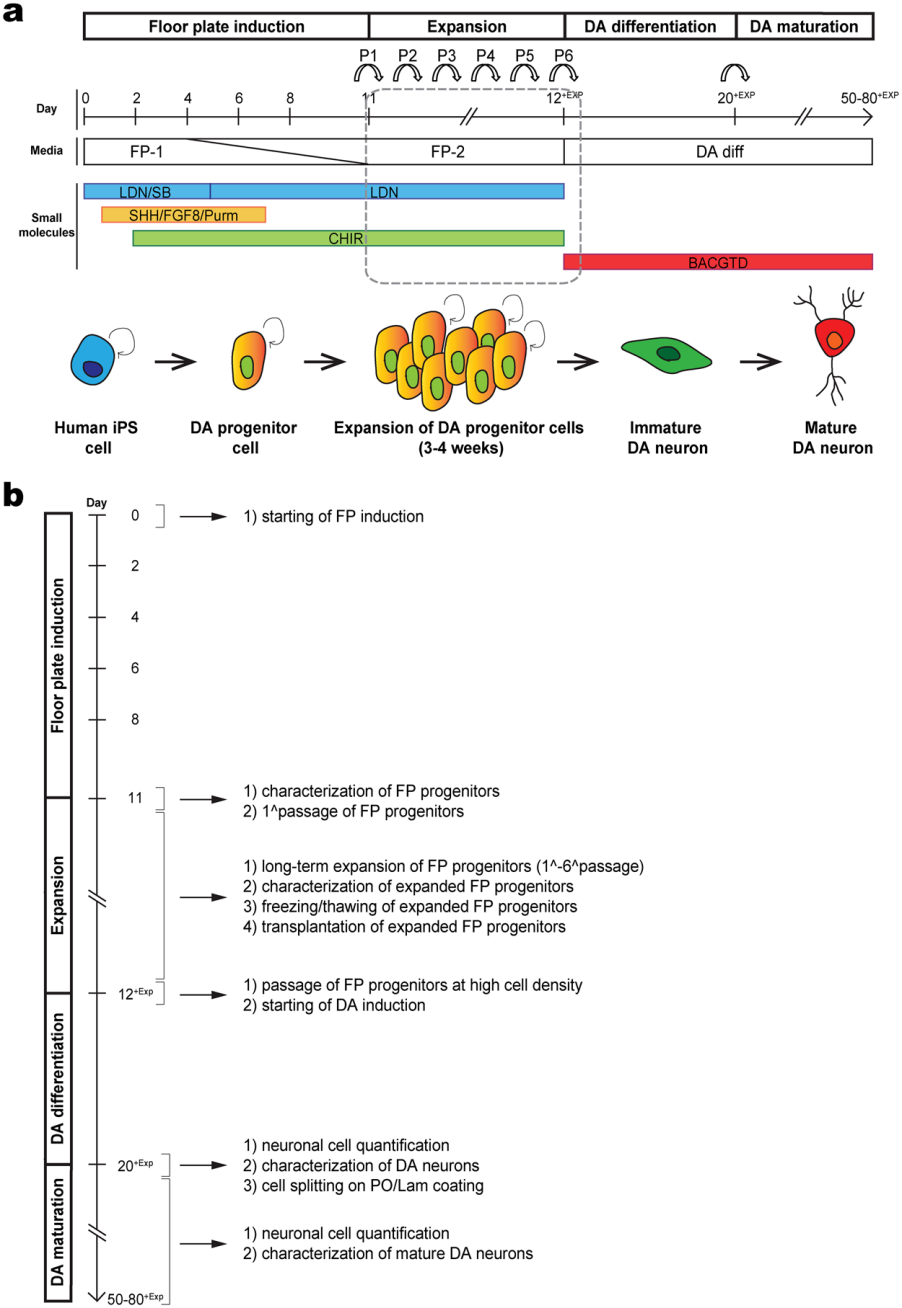
Schematic diagram for efficient expansion of *in vitro* DA neuron progenitors and final DA neuron maturation from human iPSCs. (**a**) *In vitro* DA neurons were generated using a standard mFFP protocol^11^ with some modifications. After 11 days of neuralisation and floor plate induction by adding signaling molecules such as SHH, FGF8 and CHIR-99021, mFPPs were amplified by cell passaging and maintained in culture for 3–4 weeks (6 passages in total as indicated by the arrows P1–P6). During progenitor expansion, cells were maintained into LDN-193189 and CHIR-99021. After cell expansion, differentiation of mFPPs into mature and functional DA neurons was induced up to day 50–80 (from day 12^+EXP^ to day 50–80^+EXP^). (**b**) Flow diagram of mFPP expansion and DA neuron differentiation with description of the most important steps and possible applications. FP-1, floor plate induction medium n.1; FP-2, floor plate expansion medium n.2; DA diff, DA differentiation medium; LDN, LDN-193189; SB, SB431542; Purm, Purmorphamine; CHIR, CHIR-99021; BACGTD, BDNF, ascorbic acid, cAMP, GDNF, TGF-β3, DAPT.

We developed a robust method for long-term expansion of a pure population of hiPSCs-derived mFPPs maintained in feeder-free and adherent conditions to be used as a potential source of enriched DA neuron cultures. To circumvent the heterogeneity often associated with embryoid body and neurosphere-based differentiation protocols we took advantage of the accessibility of 2D cultures to manipulate signaling pathways and bioactive molecules and factors in chemically defined serum-free media. hiPSCs were cultured on Matrigel under defined conditions in mTeSR-1 medium (see Methods). Subconfluent hiPSCs were passaged onto fresh Matrigel coated plates and cultured for 24 hours in iPSC medium to 90–100% confluency. Once confluent, the medium was changed to floor plate induction medium (n.1) and cultured for 2 days, replacing the medium every day (Fig. 1a,b). At day 3 after starting the floor plate induction with FGF8, SHH, and Purmorphamin, WNT signaling was activated by addition of the GSK3-β inhibitor CHIR-99021 to the n.1 medium. On day 5, the medium was gradually switched to floor plate expansion medium (n.2) and SB-431542 was omitted from the culture (Fig. 1a,b). By day 11 the cells were growing in an n.1/n.2 medium ratio of 1:3. At this point, cells were passaged and replated onto fresh Matrigel coated plates in n.2 medium at a density of 75 × 10^3^ cells/cm^2^. Cells were maintained in n.2 medium until 90–95% confluency (4–5 days) exchanging the medium every 2^nd^ day (Fig. 1a,b). Once the mFPPs had reached 90–95% confluency, they were passaged and replated onto fresh Matrigel coated plates at a density of 75 × 10^3^ cells/cm^2^ and cultured further in n.2 medium. We passaged and replated the mFPPs every 4–5 days for at least 6 passages and cells were cryobanked at each passage (see Methods).

**Figure 2:**
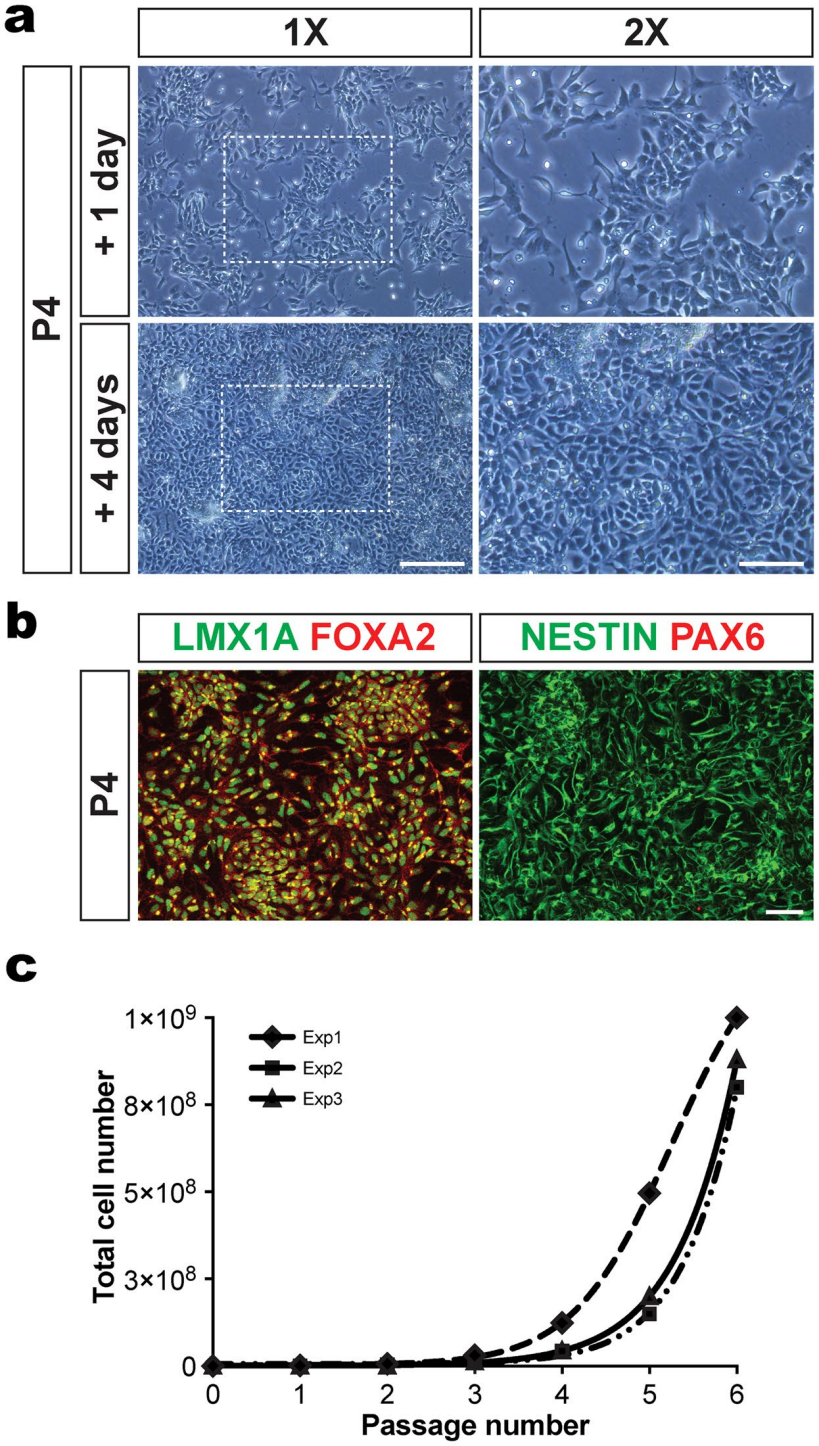
Characterization of hiPSC-derived expanded mFPPs. (**a**) Typical morphology of hiPSC-derived expanded mFPPs at passage 4 (P4) one and four days after passaging. Progenitors proliferated and reached 100% confluency within 3–4 days at each passage. (**b**) Immunocytochemical analysis of expanded mFPPs at passage 4 (P4) showed the expression for LMX1A, FOXA2 and NESTIN. No PAX6^+^ cells were observed during the expansion, demonstrating the maintenance of midbrain floor plate phenotype. (**c**) Representation of total number of mFPPs generated after 6 passages (P1–P6) in three independent experiments. At the end of the expansion (P6), 8.9 × 10^8^ ± 1 × 10^8^ progenitors were produced in total. Scale bars: 200 μm and 100 μm in **a** and 50 μm in **b**.

At day 11 of floor plate induction, 90–95% of the cells expressed the mFPPs markers LMX1A and FOXA2 and very few PAX6^+^ dorsal progenitor cells were present in the cultures, demonstrating the efficient and homogeneous mFPP induction^11^ (Fig. S1a). We examined whether the expanded mFFPs retained their characteristics with extended culture. The cells retained their morphology, forming a compact monolayer of cells that gradually became confluent (Fig. 2a). They also remained homogeneous in expression of the LMX1A and FOXA2 while PAX6^+^ cells were not detected (Figs 2b and S1b).

On average, 1.25 × 10^6^ ± 0.38 × 10^6^ mFPPs were generated at day 11 of the floor plate induction culture from 5 × 10^5^ hiPSCs. The mFPPs were dissociated and replated for >6 passages (4 weeks on average). Upon reaching confluency at the end of passage 6, 8.9 × 10^8^ ± 1 × 10^8^ hiPSC-derived mFPPs had been generated from the initial 5 × 10^5^ hiPSCs (Fig. 2c). Hence, we achieved a 720-fold increase in the number of mFPPs compared to the number at day 11 and a 1780-fold increase over the initial number of hiPSCs plated.

### Expanded mFPPs give rise to higher numbers of DA neurons at the early stage of differentiation

**Figure 3:**
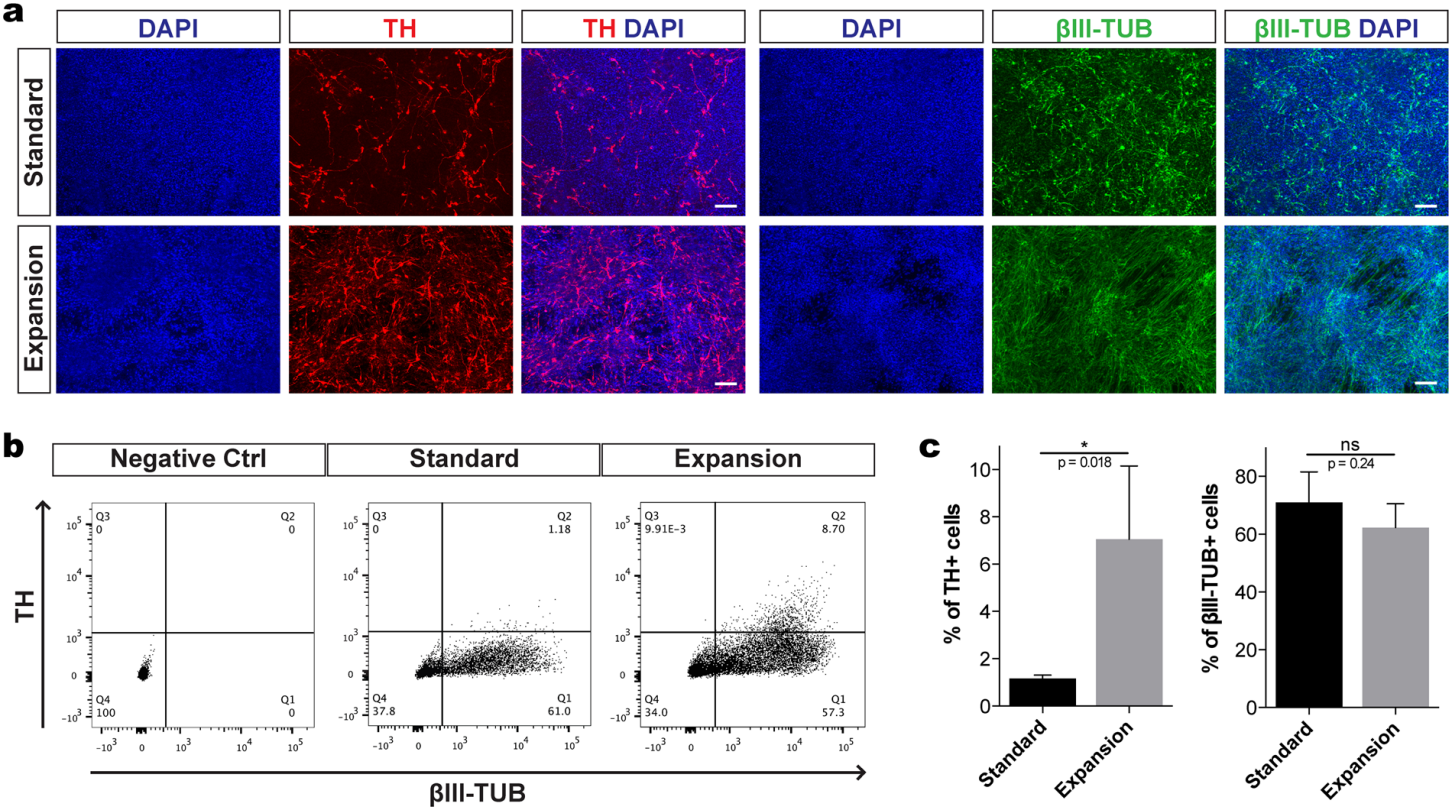
hiPSC-derived expanded mFPPs give rise to higher numbers of TH^+^ neurons at an early stage of DA neuron differentiation. (**a**) Immunocytochemical analysis of DA neurons generated by using the expansion method showed higher proportion of TH^+^ neurons compared to the standard protocol. Cells were stained with antibodies against TH and βIII-Tub (Table S1). (**b**) Dot plots of single-cell suspensions of one representative differentiation experiment using the expansion method showed higher percentages of TH^+^βIII-Tub^+^ neurons generated at day 20 compared to the standard protocol (8.70% vs 1.18%). Negative control sample was used to determine the gating parameters for cell quantification. (**c**) Comparative analysis of total positive neurons for βIII-Tub and TH markers generated at day 20 by using the standard method and the expansion protocol. Similar percentages of βIII-Tub^+^ neurons were measured using both protocols, suggesting that the progenitor expansion did not interfere with neuronal induction fate. Higher proportion of TH^+^ cells were obtained after progenitor expansion (8.06 ± 2.45%) in comparison to the standard method (1.18 ± 0.14%), resulting in a 6.83-fold increase of TH^+^ cells. Student’s t-test. Mean ± S.E.M. (n = 3–5). Scale bar: 50 μm.

We examined whether expanded mFPPs maintain their DA neuron differentiation potential compared to progenitors generated using a standard differentiation procedure. DA neuron differentiation was induced when the expanded mFPPs reached 90–100% confluency by switching the cultures to differentiation medium containing BDNF, GDNF, ascorbic acid, cAMP, β-TGF3 and DAPT (Fig. 1 and see Methods). After one week of induction (early phase of DA neuron differentiation), the expanded mFPPs cultures produced Tyrosine Hydroxylase positive (TH^+^) and βIII-Tubulin positive (βIII-Tub^+^) neurons (Fig. 3a). Quantification of differentiation by fluorescent assisted cell sorting (FACS) or microscopic analysis showed that although the proportion of βIII-Tub^+^ neurons was comparable to the standard procedure, the number of TH^+^ neurons was increased 6.8–7.4-fold (Fig. 3a–c). Hence, mFPP expansion did not interfere with general neuronal fate induction, resulting in an increase in TH^+^ neurons by day 20 (Figs 3 and S2).

### mFPP expansion increases the potential to generate mature and functional DA neurons in the late stage of differentiation

**Figure 4:**
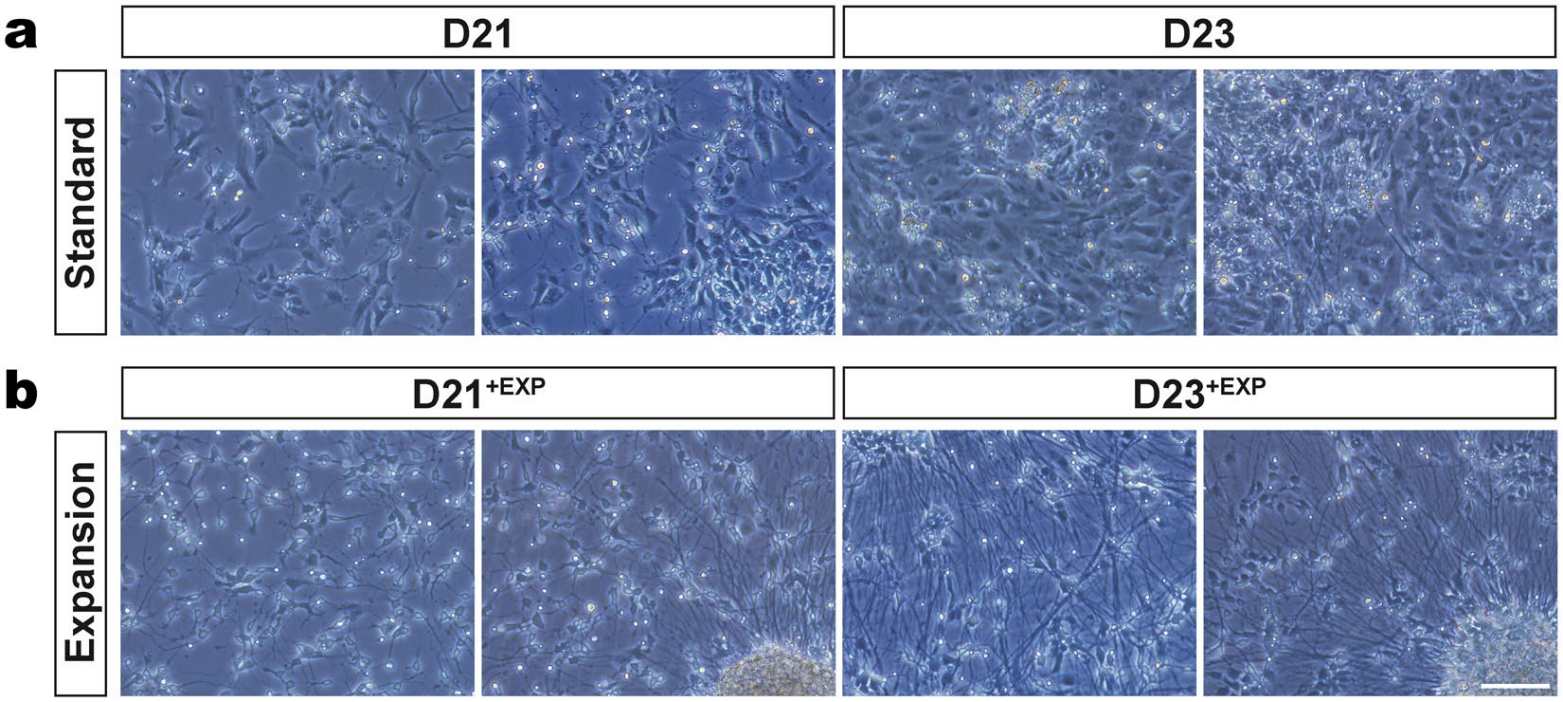
Typical cell morphology in DA neuron cell cultures obtained by using the standard and the expansion methods. (**a**) Phase image of cells after 21 (D21) and 23 days (D23) of differentiation using the standard protocol. (**b**) Phase image of cells after 21^+EXP^ (D21^+EXP^) and 23^+EXP^ (D23^+EXP^) days of differentiation using the expansion protocol. Using the expansion method, higher numbers of cells with typical neuronal morphology were generated. At D23^+EXP^, a thick net of cells with long processes could be observed, suggesting the higher efficiency of expanded progenitor-derived DA neuronal differentiation compared to the standard method. Scale bars: 100 μm.

We passaged the induced and differentiating mFPP cells on day 20, after expansion on Matrigel coated dishes, to Laminin/Fibronectin-coated plates (standard method: day 20; expansion method: day 20^+exp^) (Fig. 1). The cells from the expanded mFPP cultures and the standard cultures plated equally well. Morphologically, the mFPP cells already took on a more differentiated phenotype after one day (day 21) on Laminin/Fibronectin compared to cells cultured under standard conditions, the latter mostly had a flattened progenitor-like morphology (Figs 4 and S3). Three days after plating onto Laminin/Fibronectin-coated plates and induction of differentiation, the expanded mFPPs had generated neuron-like cells that extended processes throughout the culture dish (Figs 4 and S3). Many of the expanded mFPP progenitors condensed to form brain nucleus-like structures from which cells and processes radiated. This nucleation of the cells was less evident in the standard cultures of the same age (Figs 4 and S3). The condensation of the cells and the enhanced morphological changes were conserved over 6 passages (Fig. S3b). The differentiating neurons were maintained differentiation medium for up to 80 days in order to complete DA neuron maturation (late phase of DA neuron differentiation) (Fig. 1).

**Figure 5:**
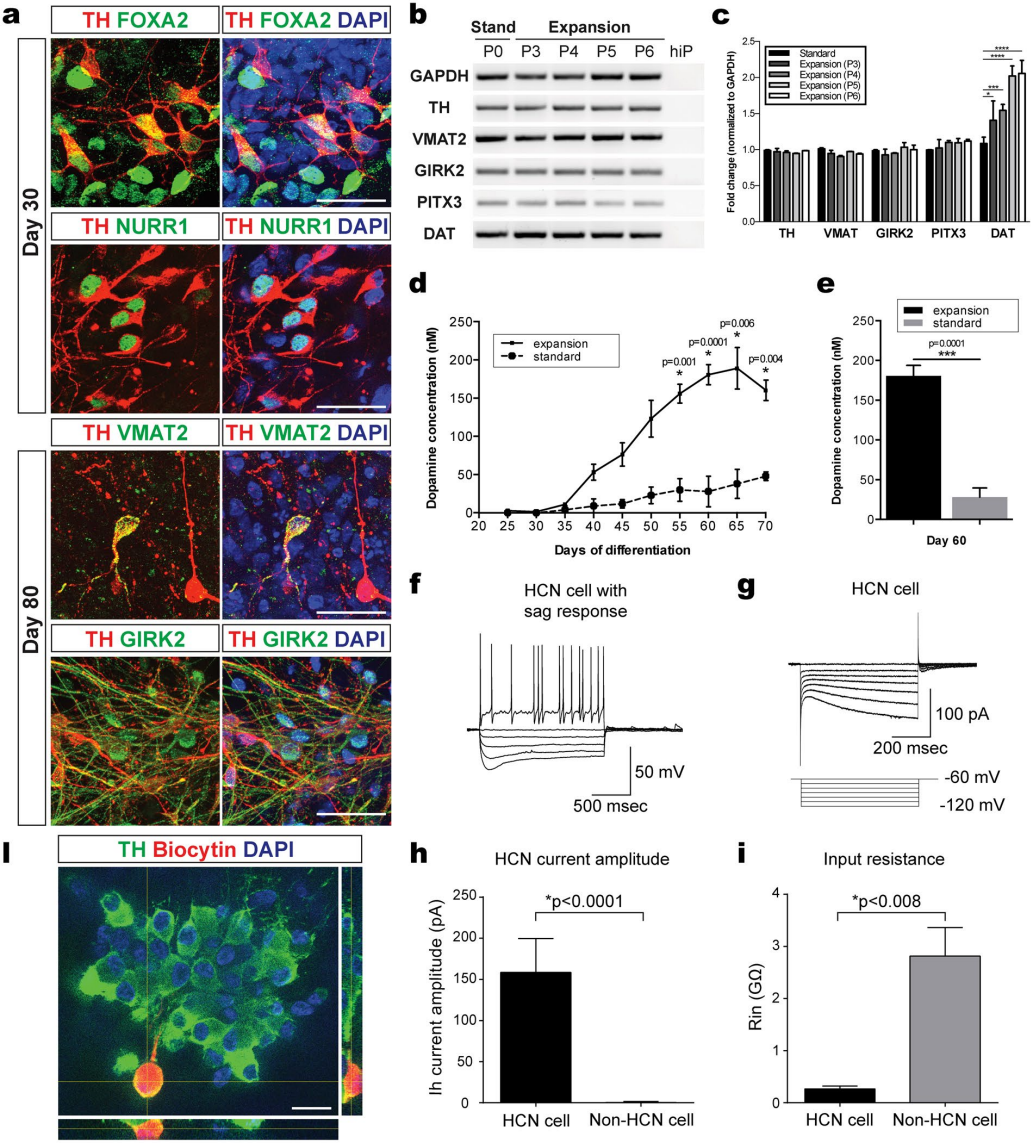
hiPSC-derived expanded mFPPs maintain their capability to differentiate into mature and functional DA neurons *in vitro*. (**a**) Immunocytochemical analysis of expanded progenitor-derived DA neurons showed the expression of mature DA neuron specific markers, such as FOXA2, NURR1, VMAT2 and GIRK2. (**b**) RT-PCR analysis revealed the expression of mature DA neuron-specific markers, such as TH, VMAT2, GIRK2, PITX3 and DAT, in DA neuron cultures after different passages of expanded progenitors (P3–P6). The image contains cropped gel images showing the respective amplicons. Images of the full gels are shown in supplemental data Figure S4 (**c**) Quantitative gene expression analysis of mature DA neuron-specific markers, TH, VMAT2, GIRK2, PITX3 and DAT, showed no different between the standard and expansion protocols. However, DAT mRNA levels were found upregulated after progenitor expansion, suggesting a higher level of maturation in expanded progenitor-derived DA neuron cultures compared to the standard procedure. ANOVA test. Mean ± S.E.M. (n = 3). (**d**) Dopamine measurement by liquid chromatography-mass spectrometry (LC-MS) in medium collected over time from DA neuron cultures generated by using either the standard or the expansion protocol. The analysis showed higher dopamine concentrations in expanded progenitor-derived DA neuron cultures compared to the standard cultures. Student’s t-test. Mean ± S.E.M. (n = 3–5). (**e**) Example of dopamine levels measured in cell cultures derived from the standard (28 ± 20 nM) and the expansion methods (180.59 ± 34.94 nM) at day 60 and day 60^+EXP^, respectively. Student’s t-test. Mean ± S.E.M. (n = 3–7). (**f**) Representative current-clamp example traces of an HCN-positive cell showing voltage sag response to hyperpolarizing current injections and marked action potentials after-hyperpolarization characteristic for DA neurons. (**g**) Representative voltage-clamp traces showing HCN currents in response to negative voltage steps. (**h**) Quantification of HCN current amplitude in HCN cells and non-HCN cells. HCN cells showed an average peak amplitude of 159 ± 41.2 pA (n = 5) versus 0.39 ± 1.10 pA measured in non-HCN cells (n = 11). Student’s t-test. Mean ± S.D. (**i**) Quantification of input resistance in HCN cells and non-HCN cells. HCN cells showed an average of 0.26 ± 0.06 GΩ (n = 5) versus 2.81 ± 0.55 GΩ detected in non-HCN cells (n = 11). Student’s t-test. Mean ± S.D. (**j**) Example of biocytin-filled HCN-positive cell, which was stained for TH expression. Immunofluorescent staining confirmed that HCN cells were effectively DA TH^+^ neurons. Scale bar: 20 μm.

We analyzed the expression of DA neuron markers by immunostaining and RT-PCR during the course of the maturation procedure and compared the standard culture-derived and expanded mFPP-derived cells (Fig. 5a,b). From day 30 of maturation, some FOXA2^+^ cells expressed the DA neuron marker TH (Fig. 5a). In addition, TH^+^ DA neurons also expressed the transcription factor NURR1 (Fig. 5a). At late stages of the differentiation procedure, the TH^+^ DA neurons expressed markers of terminal differentiation including the vesicular monoamine transporter VMAT2 and G protein-activated inward rectifier potassium channel GIRK2 (Fig. 5a). Expression of additional markers of DA neuron differentiation including the Dopamine active transporter 1 DAT and the transcription factor Pituitary homeobox 3 PITX3 was detectable in the mFPP-derived neuron cultures by RT-PCR (Figs 5b and S4). Quantitative RT-PCR revealed that the expression of mature DA neuronal markers by neurons generated from expanded mFPPs was comparable to that of DA neurons generated by the standard procedure (Fig. 5c). However, expression of DAT was higher by DA neurons derived from expanded mFPPs than those derived by the standard method (Fig. 5c). In addition, with increasing passage number the mFPPs-derived neurons expressed higher levels of DAT suggesting that they either differentiate further or quicker than neurons generated by the standard procedure. These data suggest that expansion of mFPPs has a positive effect on the differentiation and maturation of DA neurons.

An important measure of DA neuron maturation and function is their functional release of dopamine^29^. We measured the levels of dopamine in the medium of DA neurons generated either by expanded mFPPs or via the standard protocol at the same day of differentiation. Liquid chromotography-mass spectrometric (LC-MS) analysis revealed consistently higher dopamine concentrations in the medium of expanded mFPP-derived DA neurons. This increase in dopamine levels in the medium of expanded mFPP-derived neurons started at day 40 of differentiation and became most prominent at day 60 (180.6 ± 13.2 nM versus 28.0 ± 11.5 nM) (Fig. 5d,e). The 6.5-fold increase in dopamine release by expanded mFPP-derived DA neurons supports an increased activity and quicker maturation.

To further assess functional maturation of DA neurons, we performed electrophysiological recordings at day 50 of differentiation (Fig. 5f–i). Individual neurons were recorded in whole-cell voltage-clamp and current-clamp configuration in order to detect hyperpolarization-activated cyclic-nucleotide-gated channel (HCN) activity, which is a typical characteristic of mature mesencephalic DA neurons. For voltage-clamp recordings the resting membrane potential was set to −60 mV. Simultaneously, the patched neurons were filled with biocytin for subsequent morphological analysis and TH immunostaining. 31.3% of the patched neurons (5 out of 16) displayed HCN-mediated currents (Fig. 5g), important for the typical voltage sag response of DA neurons upon hyperpolarizing current injections (Fig. 5f). In those cells, the HCN-current amplitude (at −120 mV) was on average 159 ± 41.2 pA (n = 5) compared to 0.39 ± 1.10 pA measured in non-HCN neurons (n = 11) (Figs 5h and S4). HCN-current positive neurons also showed a lower input resistance (average 0.26 ± 0.06 GΩ; n = 5) compared to HCN-negative neurons (average 2.81 ± 0.55 GΩ; n = 11) (Fig. 5i). All neurons with HCN current and sag response showed TH immunoreactivity confirming their DA neuron identity (Fig. 5j). These data indicate that DA neurons generated from expanded mFPPs reached a relatively advanced level of maturity *in vitro* and showed typical expression and electrophysiological characteristics of mesencephalic DA neurons comparable to neurons isolated from the brains of mice.

### Expanded mFPPs give rise to DA neurons *in vivo*

**Figure 6:**
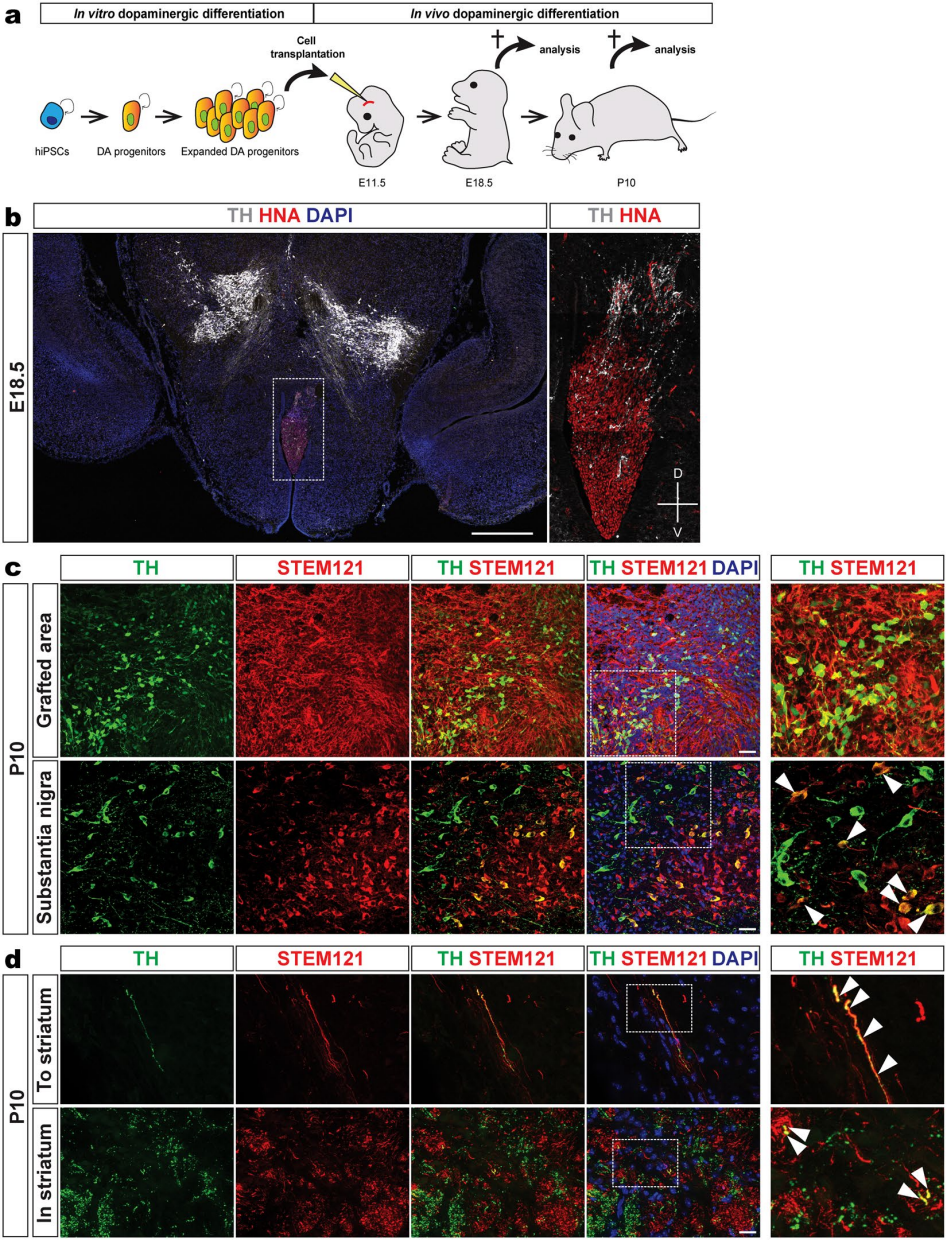
hiPSC-derived expanded mFPPs maintain their capability to differentiate into mature DA neurons *in vivo*. (**a**) Schematic representation of transplantation experiments. After mFPP expansion, cells were grafted into the mesencephalon of mouse embryos at E11.5 and their DA neuron differentiation efficiency was analyzed in embryos (E18.5) and postnatal mice (P10). (**b**) Analysis of grafted human cells in E18.5 mouse brains showed the appearance of TH^+^ cells that migrated out from the transplantation area versus the substantia nigra of the host. (**c**) Immunofluorescent analysis for TH and STEM121 markers in postnatal P10 mouse brains showed the presence of high number of human TH^+^STEM121^+^ cells in the transplantation area and their integration in the substantia nigra of the host. (**d**) Analysis of grafted human cells in P10 mouse brains revealed the presence of TH^+^STEM121^+^ neuronal processes projected to the striatum and in the striatum. Scale bars: 500 μm in **b**, 50 μm in **c** and **d**.

We examined whether expanded mFPPs retain DA neuron differentiation potential *in vivo* by grafting cells at the progenitor stage and before differentiation into the ventral mesencephalon of recipient mouse embryos *in utero* using real-time ultrasound guided microcopy (Fig. 6a). The grafted human cells were detected in the mouse brain by their expression of the human nuclear antigen (HNA) or STEM121 to detect their cellular processes (Fig. 6b–d). Upon grafting into the mesencephalon of mouse embryos at embryonic day 11.5 (E11.5), and analysis at E18.5, expanded human mFPPs that had been passaged multiple times survived, integrated into the host and generated TH^+^ DA neurons in the graft area and in the region of the substantia nigra, adjacent to the endogenous DA neurons of the host (Fig. 6b). Graft-derived cells and processes were not detected in the striatum of host mice at this stage (data not shown). By postnatal day 10 (P10), human DA TH^+^STEM121^+^ neurons were still present at the original site of the graft and some TH^+^STEM121^+^ neurons were found integrated into the substantia nigra of the host (Fig. 6c). Moreover, grafted TH^+^STEM121^+^ human neurons in the brains of P10 hosts showed neuronal processes projecting to the striatum and arborizations within the striatum (Fig. 6d). We never observed overgrowth of the grafted cells nor tumor formation suggesting the expanded mFPPs have an additional advantage over the risk of grafting undifferentiated hiPSCs which could form teratomas.

## Discussion

Here we describe and validate an important adaptation of the mFPP differentiation protocol for generating mesencephalic DA neurons. This protocol presents a number of advantages over previous protocols for generating and studying human DA neurons in culture. The mFPP protocol described previously takes advantage of the signaling networks which control midbrain formation and DA neuron differentiation^10, 11, 27, 30, 31^. Both genetic data in the mouse and *in vitro* differentiation experiments indicate that WNT signaling is important in roof plate function and mesencephalic DA neuron production *in vivo* ^32, 33^. Although mesencephalic DA neurons are generated from a ventral progenitor pool and roof plate cells are the most dorsal cells in the neural tube, both DA neurons and roof plate share expression of LMX1A^34–36^. In addition, WNT signaling has been shown to activate *LMX1A* expression by neural progenitors^37^. During brain development, Wnt1 is expressed by neural progenitors at the mid-hindbrain boundary (Isthmus) and expression extends anteriorly along the dorsal midline^38^. Wnt1 is an integral component of the Isthmus, controlling posterior midbrain and anterior hindbrain identity and development. DA neurons are born from mFPPs just anterior to the Isthmus^39^. The mFPP protocol also includes FGF8 in the initial induction steps. FGF8 is also a critical component of the Isthmus and an initiator of the organizer identity^40, 41^. SHH is well known for its ventralizing activity during development of the central nervous system^42–44^. SHH is initially expressed from the axial mesoderm and induces its own expression in floor plate cells. Hence, SHH contributes to the ventral mesencephalic fate of the neural progenitors. In addition, activation of the SHH pathway in combination with WNT promotes FOXA2/LMX1A double positive mFFPs with prominent DA neuron potential^37^. mFPP-derived DA neurons have been shown previously to be functional, and can rescue rodent and primate models of DA neuron degeneration upon grafting^10, 11^. However, one of the main challenges of the mFPP differentiation procedure has been to scale-up the production of neurons and establish reproducibility between human pluripotent cell lines. The direct differentiation of DA neurons from mFPPs is highly dependent upon the characteristics of the pluripotent cells and the efficiency of the initial floor plate determination step. Hence, we developed the culture conditions further to interrupt differentiation at the mFPP step and to expand these cells as a homogeneous population. The mFPPs continued to proliferate showing little spontaneously differentiation thus allowing for an increased period of expansion. We found that removing the activin-like receptor 5, 4 and 7 inhibitor SB-431542 and culturing the cells in n.2 medium in the presence of CHIR-99021 enhanced the expansion the maintenance of mFPPs. In contrast, presence of the activin-like receptor 2 and 3 inhibitor LDN-193189 was important for maintaining mFPPs. Addition of SHH and FGF8 did not increase mFPP expansion in n.2 medium (not shown). Previous studies have shown that the BMP4 inhibitor LDN-193189 is necessary to maintain/restrict human iPSCs to the neuroectodermal fate. On the other hand, the GSK3-β inhibitor CHIR99021 enhances survival of the FPPs at low cell density similar to its effects on embryonic stem cells to block differentiation and maintain self-renewal^45^. In addition, the Rock inhibitor Y-27632 was added to the expansion medium and promoted proliferation and maintained the morphology of FPPs in combination with CHIR99021. Y-27632 has been shown previously to enhance the survival and proliferation of dissociated human neural progenitor cells^46, 47^.

Interestingly, during extended expansion of the mFPPs, cytoplasmic expression of FOXA2 became apparent in addition to the nuclear localization. These findings support previous results indicating that *Foxa2* is rapidly inactivated *in vivo* in mouse midbrain progenitors, and is necessary but not sufficient for specification of DA neurons in human pluripotent cells^48, 49^. Hence, we think that the increase in cytoplasmic expression and reduction of FOXA2 staining in the nucleus of mFPPs does not affect the DA neuron fate commitment and differentiation efficiency *in vitro*. It is likely that as in the mouse *in vivo*, FOXA2 in DA progenitors is critical for fate specification rather than DA neuron maintenance. Moreover, we propose that FOXA2 alone is not essential to enrich for DA neurons, but other proteins such as LMX1A are likely more critical for DA neuron enrichment and differentiation.

This expansion procedure presents a number of advantages over the protocol for direct and continuous differentiation from pluripotent cells. (1) Expanding mFPPs can be passaged several times and they retain their identity thereby enabling further amplification and increasing the number of DA neurons that can be generated. (2) Expanded mFPPs can be cryopreserved at each passage to generate a biobank of homogenous cells that can be thawed as a starting material for continued differentiation. (3) Finally, the mFPP expansion procedure is robust as similar results were obtained with three independent iPSC lines, NAS2 (all of the data shown here), Invitrogen iPSC (Episomal Human iPSC Line, Life Technologies, Cat. no. A13777) and a control line F3 (Collo *et al*. in preparation) (data not shown).

In previous descriptions of DA neuron differentiation using the mFPP protocol, the number of neurons generated seemed higher than in our experiments^11, 27, 28^. These differences between the studies could be due to differences in the quantification methods or differences in the differentiation potential of the pluripotent cells used. Therefore, we made direct comparisons of the standard and mFPP expansion methods using the same iPSCs (NAS2) and quantification procedures (microscopy and FACS). To this end, we performed direct back-to-back comparisons with sister cultures differentiated via the standard direct mFPP protocol^11^. We felt it was important to test and compare the DA neurons generated from expanded mFPPs with those produced by the standard protocol not only at the transcriptome and protein level but also at the functional level. We found that the expansion period had significant beneficial effects on the degree and rate of differentiation of DA neurons in the cultures. Not only did the proportion of DA neurons increase approximately 7-fold, but the neurons also showed increased functional maturity as assessed by electrophysiology and release of dopamine. Whether this increased differentiation potential is an indication of additional selection during the expansion period or whether exposure of the cells to an extended period of mFPP induction was the major benefit remains to be established. However, the expanded mFPPs retained the ability to differentiate into DA neurons in animal models and did not show signs of transformation. Thus, taking advantage of the endogenous signaling pathways that mediate mesencephalic DA neuron differentiation, the resulting human mFPPs retain the ability to respond to endogenous cues in the midbrain tissue of host mouse embryos and to generate neurons that integrate in the host substantia nigra and project to the striatum, their correct target region in the brain. Future analysis will be required to assess their integration into the nigro-striatal circuitry and to be able to functionally rescue animal modes of DA neuron degeneration. However, based on our analyses and the similarities between the DA neurons generated by expanded mFPPs and the standard direct mFPP-derived DA neurons described previously, they most likely will also have the potential to rescue symptoms in disease models^11, 28^.

In summary, we describe and validate a protocol for the long-term expansion and propagation of mFPPs that circumvents some of the challenges faced with other, none mFPP procedures, and that provides a robust means to bank highly homogeneous cultures of DA progenitors for gene function analysis and potential drug screening. This procedure may provide a convenient and scalable source of cells for eventual cell replacement therapy.

## Methods

### Animals and husbandry

C57/BL6 mice were kept on a 12-hour day/night cycle with food and water *ad libitum* under specified pathogen free conditions in accordance with Swiss federal and Swiss veterinary office regulations. All experimental protocols were approved by the ethics commission of BaselStadt, Basel, Switzerland. All experimental procedures were carried out in accordance with Swiss Federal and Swiss veterinary office regulations under license numbers 2537 and 2538.

### Human iPSC culture and DA neuron differentiation

Human induced pluripotent stem cell (hiPSCs) lines NAS2, Invitrogen hiPSC and F3 (Dr. Ginetta Collo, manuscript submitted), were maintained on Matrigel in mTeSR-1 medium and passaged enzymatically with Accutase every 4–5 days (see online methods for details). DA neuron differentiation was induced using modifications to the FPP method^11^. Detailed description of the protocol can be found on the online version of the Methods.

### RNA isolation and RT-PCR/qPCR analysis

For RNA isolation, cells were lysed directly in Trizol (Invitrogen) reagent. RNA was isolated according to the manufacturer’s instructions. 1 μg of total RNA was used for cDNA synthesis using oligo (dT) primers and Superscript III first strand kit (Invitrogen). For RT-PCR analysis, cDNA was amplified using 5x FIREPol Master Mix (Solis BioDyne, #04-11-00125). For RT-qPCR analysis, cDNA amplification was performed using SensiFast Sybr HiROX kit (Bioline, #Bio-92020). Primer information was provided in the Supplementary Table S2.

### Fluorescence activated cell sorting for neuronal quantification

The protocol was adapted from Turac *et al*.^50^. After differentiation, cells were detached enzymatically with Accutase and a gentle resuspension was applied in order to obtain single cells. Primary antibodies used for flow cytometric analysis are shown in Supplementary Table S1. After primary antibody incubation, the cells were washed in PBS, centrifuged at 5000 rpm for 3 minutes and then resuspended in PBS and incubated with the appropriate secondary antibodies (Jackson Immunoresearch) for 30 minutes at room temperature on a shaker in the dark. Finally, cells were washed in PBS and centrifuged at least 3 times, resuspend in 2% FBS in PBS and filtered through a 40 μm cell sieve (Miltenyi Biotec). The cells were sorted on a fluorescence-activated cell sorter FACS Canto II (BD Biosciences) and analyzed using FACSDiva software (BD Biosciences). Data were additionally analyzed and presented using FlowJo software.

### Quantification and statistical analysis

Stained coverslips or sections were analyzed with fixed photomultiplier settings on a Zeiss Observer with Apotome (Zeiss) or Zeiss LSM510 confocal microscope (Zeiss). Data are presented as averages of a minimum of three independent differentiation experiments. Statistical comparisons were determined by two-tailed unpaired Student’s *t*-test, Kruskal-Wallis with Dunn post-hoc test and two way ANOVA test for cross comparison of 3 and more data sets. Significance was established at ^*^ *p* < *0.05*. In all graphs, deviance from mean was displayed as standard error of the mean, SEM.

## Electronic supplementary material

Supplementary information
