# Supplementary material for: Expansion of human midbrain floor plate progenitors from induced pluripotent stem cells increases dopaminergic neuron differentiation potential
Source: Sci Rep. 2017 Jul 20;7:6036. doi: 10.1038/s41598-017-05633-1 (PMC5519680; doi:10.1038/s41598-017-05633-1)
Supplement: Supplementary file 1 — Supplementary information [file 41598_2017_5633_MOESM1_ESM.pdf]

Expansion of human midbrain floor plate progenitors from induced pluripotent stem cells increases dopaminergic neuron differentiation potential

Authors

Stefania Fedele[1], Ginetta Collo[1,2], Katharina Behr[3], Josef Bischofberger[3], Stephan Müller[4], Tilo Kunath[5], Klaus Christensen[4], Anna Lisa Gündner[4], Martin Graf[4], Ravi Jagasia[4], Verdon Taylor[1]

SUPPLEMENTARY FIGURES

Supplementary Figure S1

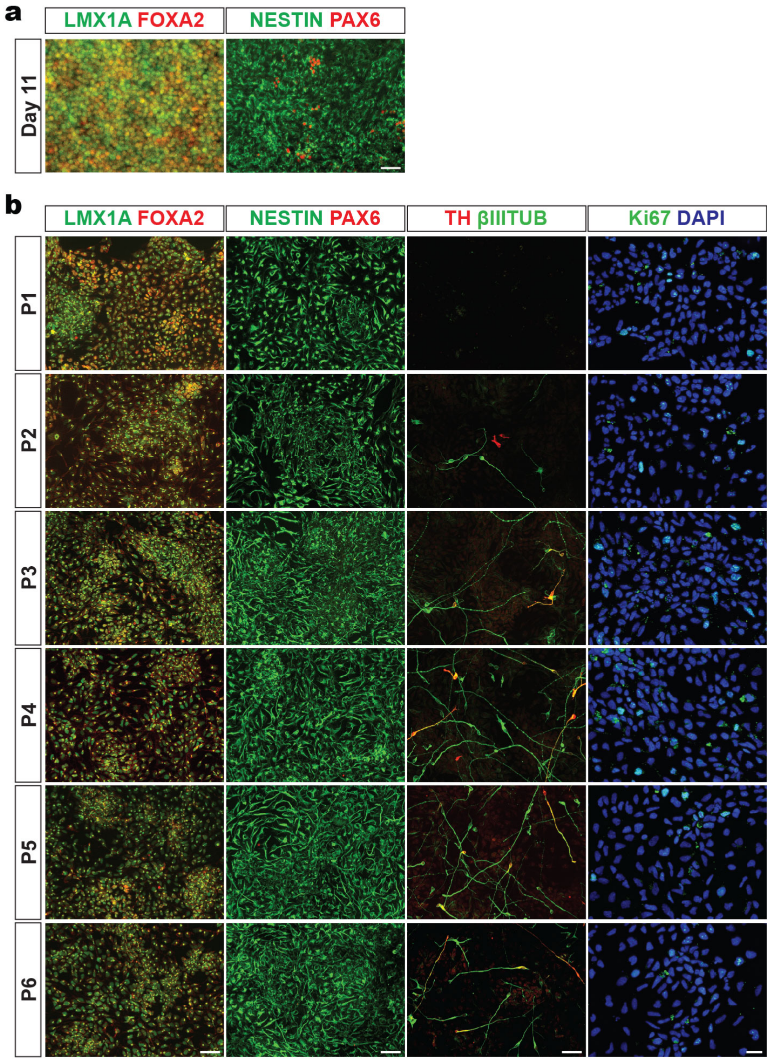

Supplementary Figure S2

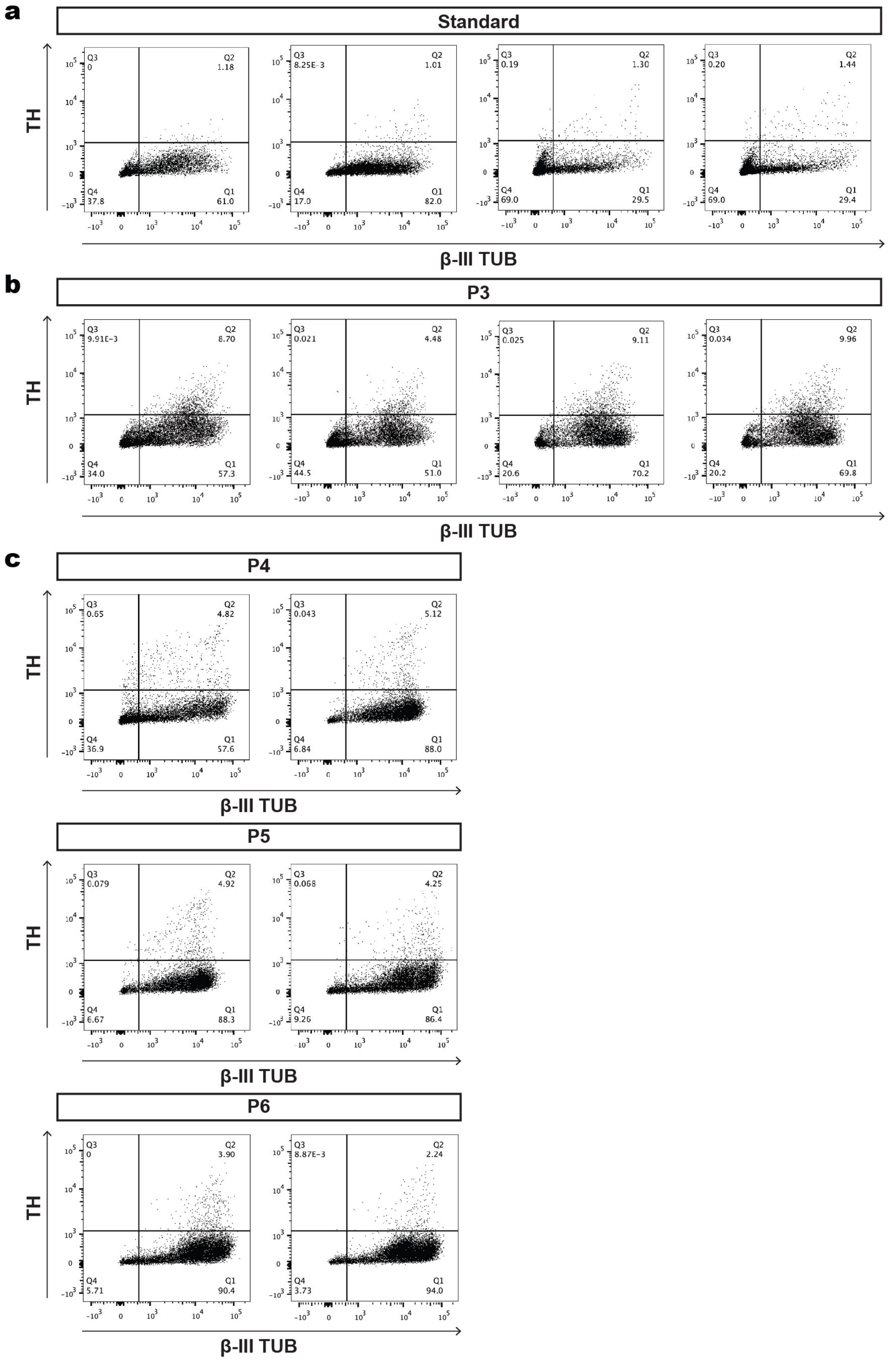

Supplementary Figure S3

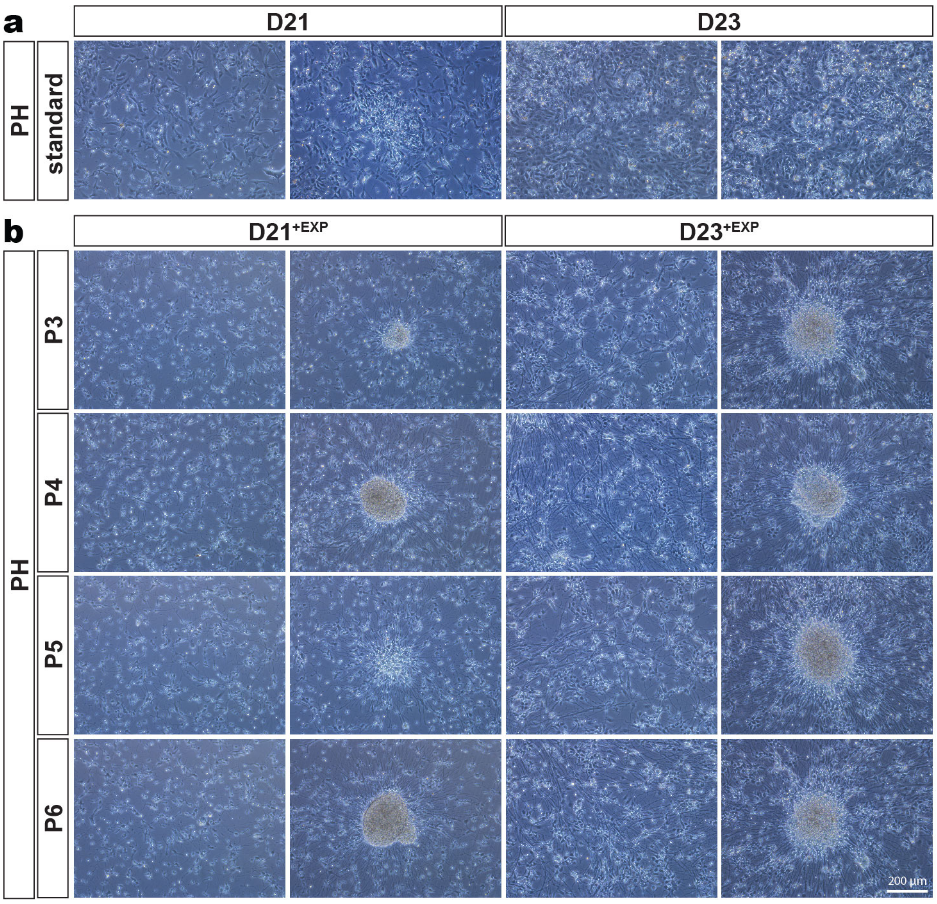

Supplementary Figure S4

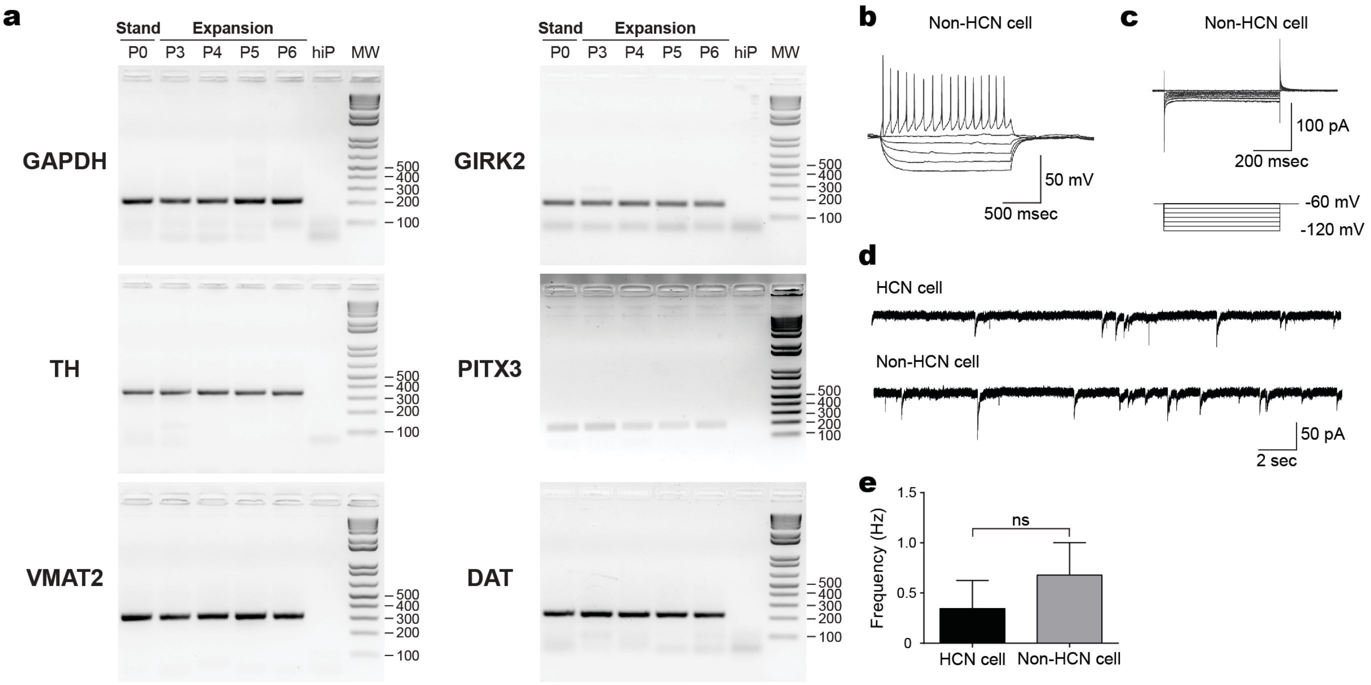

SUPPLEMENTARY TABLES

Supplementary table S1. Primary antibodies.

Primary antibody	Isotype	Dilution	Manufacturer	Application
FOXA2	Goat	1:600	Santa Cruz, cat. no. SC-9187	IF
HNA	Mouse	1:200	Millipore, cat. no. MAB1281	IF
LMX1A	Rabbit	1:1000	Sigma, cat. no. HPA030088	IF
NURR1	Mouse	1:100	Perseus Proteomics, cat. no. PP-N1404-00	IF
PAX6	Rabbit	1:600	Covance, cat. no. PRB-278-P	IF
STEM121	Mouse	1:1000	Clonotech, cat. no. Y40410	IF
TH	Rabbit	1:500	Santa Cruz, cat. no. SC-14007	IF
TH	Mouse	1:500	Millipore, cat. no. MAB318	IF
TH	Mouse	1:200	Pel-Freez, cat. no. P40101-0	FACS
VMAT2	Rabbit	1:50	Millipore, cat. no. AB1598P	IF
β III-Tubulin	Mouse	1:600	Sigma, cat. no. T8660	IF, FACS (1:150)

Supplementary table S2. Primers used for RT-PCR and RT-qPCR.

Gene name	Forward	Reverse
DAT	ACAGAGGGGAGGTGCGCCAGTTCACG	ACGGGGTGGACCTCGCTGCACAGATC
GAPDH	TCGGAGTCAACGGATTTGGT	CCTGGAAGATGGTGATGGGA
GIRK2	GCTACCGGGTCATCACAGAT	ACTGCATGGGTGGAAAAGAC
PITX3	GTGGGTGGAGAGGAGAACAA	TTCCTCCCTCAGGAAACAATG
TH	GTCCCCTGGTTCCCAAGAAAAGT	TCCAGCTGGGGGATATTGTCTTC
VMAT2	CTTTGGAGTTGGTTTTGC	GCAGTTGTGATCCATGAG

SUPPLEMENTARY FIGURE LEGENDS

Supplementary Figure S1. Characterization of hiPSC-derived expanded mFFPs. (a) Immunocytochemical analysis of mFPPs at day 11 for LMX1A and FOXA2. A pure and homogenous population of mFPPs LMX1A^{+}FOXA2^{+} was generated after 11 days, indicating the high efficiency of floor plate induction protocol. (b) Immunocytochemical analysis of expanded mFPPs at each passage (from P1 to P6) for LMX1A, FOXA2, NESTIN, TH, βIII-Tub, PAX6 and Ki-67. Persistent presence of LMX1A^{+}FOXA2^{+} cells and absence of PAX6^{+} cells during the expansion demonstrated the maintenance of midbrain floor plate phenotype. Few TH^{+}βIII-Tub^{+} cells were observed starting from the second passage (P2). Proliferating cells were stained with the antibody against Ki-67, a cellular marker for proliferating cells. Scale bar: 50 μm.

Supplementary Figure S2. hiPSC-derived expanded mFPPs give rise to higher number of TH^{+} neurons at early stage of DA neuron differentiation. Dot plots of single-cell suspensions of 4 independent differentiation experiments using the standard method (a) and the expansion method (b, c). (b) Cell quantification of TH^{+}βIII-Tub^{+} neurons generated after three passages of progenitor expansion (P3) in 4 independent differentiation experiments using the expansion method. (c) Cell quantification of TH^{+}βIII-Tub^{+} neurons generated after later passages of progenitor expansion (P4, P5, P6) in 2 independent differentiation experiments each passage using the expansion method.

Supplementary Figure S3. Typical cell morphology in DA neuron cultures obtained by using the standard and the expansion methods. (a) Phase image of cells after 21 (D21) and 23 days (D23) of differentiation using the standard protocol. Some cells with neuronal morphology and many flat and large cells were visualized using a light microscope. Cells reached the confluence 2 days after, suggesting the high rate proliferation of these cell cultures. (b) Phase image of cells after 21^{+EXP} (D21^{+EXP}) and 23^{+EXP} (D23^{+EXP}) days of differentiation using the expansion protocol. Higher number of cells with typical neuronal morphology was generated after progenitor expansion compared to the standard method. One day after cell passaging at day 20 (D21^{+EXP}), clumps were highly composed of cells with typical neuronal morphology, which projected their processes outside of clumps and formed a

complex network with surrounding cells. At D23^{+EXP} a thick net of cells with long neuronal processes could be observed, suggesting the higher efficiency of expanded progenitor-derived DA neuronal differentiation compared to the standard method. Scale bar: 200 µm.

Supplementary Figure S4. **Expanded mFPP-derived DA neurons show typical electrophysiological characteristics of mature mesencephalic DA neurons.** (a) Images of the RT-PCR analysis shown in Figure 5b. (b) Current-clamp example traces of a non-HCN cell showing no voltage sag response upon hyperpolarizing current injections. (c) Representative voltage-clamp traces of a non-HCN cell´s current response to negative voltage steps. (d) Example traces of spontaneous synaptic currents in HCN and non-HCN cells. (e) Frequency of spontaneous synaptic currents in HCN and non-HCN cells. HCN cells showed an average of 0.35 ± 0.28 Hz (n=3) versus 0.68 ± 0.32 Hz measured in non-HCN cells (Student´s t-test. Mean ± S.D. n=5). Notably, 5 cells over a total of 16 were HCN cells, suggesting that 31.25% cell culture was composed of functional DA neurons (n=3-5).

METHODS

Human iPSC culture

Three different induced pluripotent stem cell (hiPSCs) lines, called as NAS2 (provided by Prof. Tilo Kunath), Invitrogen (provided by Holfmann-La Roche) and F3 (provided by Dr. Ginetta Collo), were maintained on Matrigel (BD, #354277) in mTeSR-1 medium (Stem Cell Technologies, mTeSR1 Basal Medium #05850 and mTeSR1 5x supplement #05851). hiPSCs were passaged enzymatically with Accutase (StemCell Technologies, #07920) every 4-5 days and plated in the presence of 10 µM Y27632 Rock inhibitor (Miltenyi Biotec, #130-104-169).

In vitro expansion of floor plate midbrain progenitors and DA neuron differentiation

For DA neuron differentiation, hiPSCs were disaggregated using Accutase for 2 minutes, centrifuged at 800 rpm for 4 minutes and plated on Matrigel-coated multiwells in the presence of Rock inhibitor at the density of 200.000 cells/cm^2. When the cells reached a confluent state, DA neuron differentiation was induced according to the protocol of Kriks et al. with some modifications. Briefly, from day 0 to day 5, SB431542 (10 µM; Stemgent, #040074) and LDN193189 (100 nM; Miltenyi Biotec, #130-096-226) were added into the floor plate induction medium n.1. SHH-C24 (100 ng/mL; Miltenyi Biotec, #130-095-727), FGF8 (100 ng/mL; Miltenyi Biotec, #130-095-740) and Purmorphamine (2 µM; Miltenyi Biotec, #130-104-465) were present in the floor plate induction medium n.1 and floor plate expansion medium n.2 from day 1 to day 7. CHIR99021 (3 µM; Miltenyi Biotec, #130-103-926) was added at day 3 until day 11. On day 5 of differentiation, the floor plate induction medium n.1 was gradually shifted to the floor plate expansion medium n.2. Cells were maintained on Matrigel in LDN193189 and CHIR99021 from day 7 to day 11. At day 11, DA cell progenitors were split on Matrigel at the density of 75.000 cells/cm^2 and maintained in the floor plate expansion medium n.2 supplemented with 100 nM LDN193189, 3 µM CHIR99021 and 10 µM Y27632 Rock inhibitor. The medium was changed every second day with the addition of the three small molecules. Once 90-95% of confluence was reached, DA progenitor cells were passaged enzymatically with Accutase, centrifuged at 800 rpm for 4 minutes, counted and plated on new Matrigel-coated multiwells at the density of 75.000 cells/cm^2 in the presence 100 nM LDN193189, 3 µM CHIR99021 and 10 µM Y27632 Rock inhibitor. Cells were

expanded and maintained in the floor plate expansion medium n.2 supplemented with LDN193189, CHIR99021 and Y27632 Rock inhibitor for more than one month for maximum 6 passages as average. After long-term expansion of progenitors, cells were transferred on fresh Matrigel-coated multiwells to induce the DA neuron fate in the DA differentiation medium supplemented with BDNF (20 ng/mL; Miltenyi Biotec, #130-096-286), GDNF (20 ng/mL; Miltenyi Biotec, #130-098-449), TGF-β3 (1 ng/mL; Miltenyi Biotec, #130-094-007), ascorbic acid (200 μM; Sigma, A4034), dibutyryl cAMP (0.5 mM; Sigma, D0627) and DAPT (10 μM; Tocris, #2634). After 7 days in the complete differentiation medium, cells were plated at 75.000 cells/cm^2 on poly-L-ornithine/laminin coated-coverslips or multiwells (15 μg/mL poly-L-ornithine – Sigma, #3655; 20 μg/mL laminin – Roche, #11243217001). Half volume of DA differentiation medium was changed every day and the cells were cultured for the final DA maturation until day 50-60 according to experimental goals.

Floor plate induction medium n.1

Floor plate induction medium n.1 contains KO DMEM supplemented with 15% KO serum replacement, 2 mM Glutamax, 1x mM MEM non-essential amino acids, 50 U/ml penicillin, 50 mg/ml streptomycin and 55 μM 2-mercaptoethanol. To prepare 500 ml floor plate induction medium n.1, mix 409.5 ml KO DMEM with 75 ml KO serum replacement, 5 ml Glutamax, 5 ml non-essential amino acids and 5 ml penicillin/streptomycin. Filter with a bottle-top filter (0.2 μm), add 500 μl 2-mercaptoethanol. Use n.1 within one week and store at 4°C in the dark.

Floor plate expansion medium n.2

Floor plate expansion medium n.2 contains DMEM/F12 with Hepes buffer supplemented with N2 supplement (1x final concentration), 2 mM Glutamax, 50 U/ml penicillin and 50 mg/ml streptomycin. To prepare 50 ml floor plate expansion medium n.2, mix 48.5 ml DMEM/F12 with Hepes buffer with 500 μl N2 supplement 1x, 500 μl Glutamax, 500 μl penicillin/streptomycin. Filter with a 0.22-μm pore size filter thought a plastic disposable syringe. Use n.2 within one week and store at 4°C in the dark.

Progenitor expansion medium

Progenitor expansion medium contains DMEM/F12 with Hepes buffer supplemented with N2 supplement (1x final concentration), 2 mM Glutamax, 50 U/ml penicillin and 50 mg/ml

streptomycin. To prepare 50 ml progenitor expansion medium, mix 48.5 ml DMEM/F12 with Hepes buffer with 500 µl N2 1x, 500 µl Glutamax and 500 µl penicillin/streptomycin. Filter with a 0.22-µm pore size filter thought a plastic disposable syringe. Use progenitor expansion medium within one week and store at 4°C in the dark.

Progenitor freezing medium

Progenitor freezing medium contains DMEM/F12 with Hepes buffer supplemented with N2 supplement (1x final concentration), 2 mM Glutamax, 50 U/ml penicillin, 50 mg/ml streptomycin and 10% DMSO. To prepare 10 ml progenitor freezing medium, mix 8.7 ml DMEM/F12 with Hepes buffer with 100 µl N2 supplement 1x, 100 µl Glutamax, 100 µl penicillin/streptomycin and 1 ml DMSO. Store progenitor freezing medium at 4°C and use on the same day of preparation.

DA neuron differentiation medium

DA neuron differentiation medium contains Neurobasal medium supplemented with B27 (1x final concentration), 2 mM Glutamax, 50 U/ml penicillin and 50 mg/ml streptomycin. To prepare 50 ml DA differentiation medium, mix 48 ml Neurobasal medium with 1 ml B27 1x, 500 µl Glutamax and 500 µl penicillin/streptomycin. Filter with a 0.22-µm pore size filter thought a plastic disposable syringe. Use DA differentiation medium within one week and store at 4°C in the dark.

Immunocytochemistry and microscopy

For immunofluorescence staining, cells were fixed in 4% paraformaldehyde (PFA) for 10 minutes, rinsed with PBS and then incubated with blocking solution (10% normal donkey serum, 0.1%-0.5% Triton X-100, 1% BSA) 1 hour at room temperature. In the same blocking buffer, the cells were then immunostained overnight at 4°C with the desirable primary antibodies (Supplementary Table S1). After additional rinsing with PBS, corresponding fluorochrome-conjugated secondary antibodies (Jackson Immunoresearch) were added for 1 hour at room temperature. Cells were then counterstained with DAPI (1 µg/mL) and embedded in mounting medium containing diazabicyclo-octane (DABCO; Sigma). Images were acquired using fixed photomultiplier settings on a Zeiss Observer with Apotome (Zeiss)

or a Zeiss LSM510 confocal microscope with ImageJ 1.46r or Photoshop CS6 (Adobe) software.

DA release measured by LC/MS-MS SRM

Human iPS cell-derived DA neurons were differentiated on poly-ornithine/laminin coated-coverslips and half volume of medium was changed every day. At different days of DA differentiation, 1-2 mL medium were collected and the dopamine release in cell medium was stabilized with 1:100 dilution of 40% phosphoric acid (H_3PO_4). Collected medium samples were immediately stored at -80°C before the analysis. The dopamine concentration was determined by liquid chromatography/mass spectrometry (LC/MS-MS SRM) in positive ion mode. At desirable time, samples were thawed and 0.5 mL medium was mixed with 5 μL of internal standard and 0.5 mL distilled water. Samples were added to a sample preparation column for catecholamines (ClinRep/RECIPE). Samples were mixed for 10 minutes on an overhead shaker and afterwards they were centrifuged for 1 minute at 1000 rpm and washed in a washing solution for three times. 120 μL eluting reagent for catecholamines (ClinRep/RECIPE) was added to the column, mixed for 5 minutes at 850 rpm on a shaker and then it was centrifuged for 1 minutes at 1000 rpm. Flow-throughs were transferred into a vial and were used for the analysis. Calibration standard curve was prepared in 0.01 M HCl with serial dilution from 0 μM to 10 μM.

Electrophysiological analysis of differentiated DA neurons

For whole-cell voltage-clamp and current-clamp electrophysiological recordings, coverslips with differentiated neurons (day 50^{+EXP}) were continuously superfused with ACSF containing (in mM): 126 NaCl, 25 D-Glucose, 10 HEPES, 25 $NaHCO_3$, 2.5 KCl, 1 NaH_2PO_4, 2 $CaCl_2$ and 1 $MgCl_2$. Patch pipettes (2.5-4 MΩ) were pulled from borosilicate glass tubing with 2 mm outer diameter and 0.5 mm wall thickness (Hilgenberg, Germany) and filled with internal solution containing (in mM): 80 KCl, 62.5 potassium gluconate, 10 HEPES, 10 EGTA, 2 $MgCl_2$, 2 Na_2ATP, 1 sodium phosphocreatine and 0.3 Na-GTP (pH was adjusted to 7.3 with KOH). For biocytin filling of patched neurons, the internal solution was supplemented with 2.5 mg biocytin per mL. All recordings were performed at 22-25 °C.

Voltage signals and currents were measured with a Multiclamp 700B amplifier (Molecular Devices), filtered at 2-10 kHz and digitized with 20 kHz using a 16-bit CED Power 1401 interface (Cambridge Electronic Design). Data acquisition and analysis were achieved using a custom software (FPulse, U. Fröbe, Physiological Institute, Freiburg) running under IGOR Pro 6.31 (WaveMetrics) and the open source software Stimfit (http://code.google.com/p/stimfit, C. Schmidt-Hieber, University College London). The holding potential for voltage and current clamp recordings was set to -60 mV.

Transplantation of expanded progenitors in mouse brain

Expanding mFPPs were dissociated in Accutase (StemCell Technologies, #07920), centrifuged, filtered, counted and suspended to a final concentration of 100.000-200.000 cells/µL. A maximum volume of 10-20 nL were injected into the mesencephalon of E11.5 mouse embryos (C57/BL6 mice). For histological analysis in mouse embryos, embryos were collected at E18.5, and the brains were fixed in 4% paraformaldehyde (PFA) solution in 0.1 M phosphate buffer overnight. Brains were cryoprotected in a 30% sucrose solution in 0.1 M phosphate buffer for 24 h, embedded and frozen over dry ice in OCT (TissueTEK) and then cut as 30 µm sections by cryostat (Leica) and collected on glass. In the study of adult brains, mice were deeply anesthetized by injection of a ketamine/xylazine/acepromazine solution (150 mg, 7.5 and 0.6 mg per kg body weight, respectively). Animals were perfused with ice-cold 0.9% saline followed by 4% paraformaldehyde (PFA) in 0.1 M in phosphate buffer. Brains were isolated and post-fixed overnight in 4% paraformaldehyde in 0.1 M phosphate buffer, and then cryoprotected with 15% sucrose overday followed by 30% sucrose in phosphate buffer at 4°C overnight. Afterwards brains were embedded and frozen in OCT (TissueTEK) and then cut as 30 µm floating sections by cryostat (Leica). Free-floating coronal sections were stored at -20°C in antifreeze solution until use.

For immunostaining, sections were blocked at room temperature for one hour with blocking solution of 10% normal donkey serum (Jackson Immunoresearch), 0.1-0.5% Triton X-100, 1% BSA in PBS. Next, they were incubated overnight at 4°C with the primary antibodies (Supplementary Table S1) diluted in the same blocking solution. Sections were washed in PBS and incubated at room temperature for 1 hour with the appropriate secondary antibodies. When signal amplification was needed, sections were washed and incubated for 1

h at room temperature in streptavidin-FITC (Jackson ImmunoResearch; 1:300) and counterstained with DAPI (1 μg/ml). Stained sections were embedded in mounting medium containing diazabicyclo-octane (DABCO; Sigma) and images were acquired with Zeiss LSM510 confocal microscope (Zeiss).

Fluorescence activated cell sorting for neuronal quantification

The protocol was adapted from Turac et al.[44]. After differentiation, cells were detached enzymatically with Accutase and a gentle resuspension was applied in order to obtain single cells. Cells were then centrifuged at 800 rpm for 4 minutes and immediately fixed in 2% paraformaldehyde (PFA) for 15 minutes at room temperature on a shaker. After fixation, fixed cells were washed in PBS, centrifuged at 5000 rpm for 3 minutes and resuspended in permeabilization buffer (0.7% Tween-20) for 15 minutes at room temperature on a shaker. Then, they were washed, centrifuged and resuspended in 1% BSA, 10% NDS, 0.5% Tween-20 buffer in PBS with the primary antibodies for one hour at room temperature on a shaker. Primary antibodies used for flow cytometry analysis are shown in Supplementary Table S1. In the end of primary antibody incubation, the cells were washed in PBS, centrifuged at 5000 rpm for 3 minutes and then resuspended in PBS with the appropriate secondary antibodies (Jackson Immunoresearch) for 30 minutes at room temperature on a shaker in the dark. Finally, cells were washed in PBS and centrifuged for at least 3 times, resuspend in 2% FBS in PBS and filtered through a 40 μm cell sieve (Miltenyi Biotec). They were sorted on a fluorescence-activated cell sorter FACS Canto II (BD Biosciences) and analyzed using FACSDiva software (BD Biosciences). Data were additionally analyzed and presented using FlowJo software. For each cell preparation, a negative control (minimal number of 100.000 cells) was incubated just in the presence of the secondary antibodies. For stained cells, a minimal range of 300.000-500.000 cells was analyzed by flow cytometry. All analyses and sorts were repeated at least three times. A 70 μm ceramic nozzle (BD Biosciences), sheath pressure of 20–25 pounds per square inch (PSI), and an acquisition rate of 1.000–3.000 events per second were used. Antibodies used for flow cytometry analysis were firstly titrated.
